# Reduced Levels of H_2_S in Diabetes-Associated Osteoarthritis Are Linked to Hyperglycaemia, Nrf-2/HO-1 Signalling Downregulation and Chondrocyte Dysfunction

**DOI:** 10.3390/antiox11040628

**Published:** 2022-03-25

**Authors:** María Piñeiro-Ramil, Elena F. Burguera, Tamara Hermida-Gómez, Beatriz Caramés, Natividad Oreiro-Villar, Rosa Meijide-Faílde, Francisco J. Blanco, Carlos Vaamonde-García

**Affiliations:** 1 Tissue Engineering and Cellular Therapy Group, Biomedical Research Institute of A Coruña (INIBIC), Centro de Investigaciones Científicas Avanzadas (CICA), Departamento de Fisioterapia, Medicina y Ciencias Biomédicas, Facultad de Fisioterapia, Universidade da Coruña, 15006 A Coruña, Spain; maria.pramil@udc.es (M.P.-R.); rosa.meijide.failde@udc.es (R.M.-F.); 2 Grupo de Investigación en Reumatología, Instituto de Investigación Biomédica de A Coruña (INIBIC), Complexo Hospitalario Universitario de A Coruña (CHUAC), SERGAS, 15006 A Coruña, Spain; elena.fenandez.burguera@sergas.es (E.F.B.); tamara.hermida.gomez@sergas.es (T.H.-G.); beatriz.carames.perez@sergas.es (B.C.); natividad.oreiro.villar@sergas.es (N.O.-V.); 3 Grupo de Investigación en Reumatología y Salud, Centro de Investigaciones Científicas Avanzadas (CICA), Departamento de Fisioterapia, Medicina y Ciencias Biomédica, Facultad de Fisioterapia, Universidade da Coruña, 15006 A Coruña, Spain; 4 Centro de Investigación Biomédica en Red, Bioingeniería, Biomateriales y Nanomedicina (CIBER-BBN), 28029 Madrid, Spain; 5 Grupo de Investigación en Reumatología y Salud, Centro de Investigaciones Científicas Avanzadas (CICA), Departamento de Biología, Facultad de Ciencias, Universidade da Coruña, 15071 A Coruña, Spain

**Keywords:** hydrogen sulphide, osteoarthritis, type 2 diabetes, chondrocytes, inflammation, nuclear factor-erythroid 2-related factor-2, heme oxygenase-1

## Abstract

Different findings indicate that type 2 diabetes is an independent risk factor for osteoarthritis (OA). However, the mechanisms underlying the connection between both diseases remain unclear. Changes in the balance of hydrogen sulphide (H_2_S) are thought to play an important role in the pathogenesis of diabetes and its complications, although its role is still controversial. In this study, we examined the modulation of H_2_S levels in serum and chondrocytes from OA diabetic (DB) and non-diabetic (non-DB) patients and in cells under glucose stress, in order to elucidate whether impairment in H_2_S-mediated signalling could participate in the onset of DB-related OA. Here, we identified a reduction in H_2_S synthesis in the cartilage from OA-DB patients and in cells under glucose stress, which is associated with hyperglycaemia-mediated dysregulation of chondrocyte metabolism. In addition, our results indicate that H_2_S is an inductor of the Nrf-2/HO-1 signalling pathway in cartilage, but is also a downstream target of Nrf-2 transcriptional activity. Thereby, impairment of the H_2_S/Nrf-2 axis under glucose stress or DB triggers chondrocyte catabolic responses, favouring the disruption of cartilage homeostasis that characterizes OA pathology. Finally, our findings highlight the benefits of the use of exogeneous sources of H_2_S in the treatment of DB-OA patients, and warrant future clinical studies.

## 1. Introduction

Osteoarthritis (OA) is the most common chronic joint pathology, showing a prevalence of 10–20% among the population aged over 50 years [1,2]. The pathogenesis of OA involves cartilage degradation, subchondral bone sclerosis, and synovial inflammation, which causes pain and, eventually, the loss of articular function [3]. Although the aetiology of OA is still unclear, it is widely accepted that a shift in cartilage metabolism towards catabolic pathways may contribute to OA development [3,4]. Moreover, growing evidence indicates that reactive oxygen species (ROS) production is increased in OA, which raises oxidative stress levels and may also play a role in the pathogenesis of this disease.

Epidemiological studies have revealed that ageing is only one of the multiple risk factors for OA [5]. In fact, several clinical OA phenotypes can be discriminated based on risk factors such as ageing, trauma, obesity, or metabolic syndrome [6]. Regarding the latter, the association of metabolic diseases (obesity, diabetes, and insulin resistance, dyslipidaemia, and hypertension) and OA is the most recently studied and leads to the concept of systemic regulation of joint tissues [6]. For instance, the association between obesity and OA extends beyond weight-bearing joints, suggesting that this link is not solely based on mechanical factors. Epidemiological findings have also shown that type 2 diabetes (DB), the most common form of diabetes, predicts the development of severe OA and the presence of radiographic and symptomatic disease independently of age and body mass index (BMI) [7,8]. All together, these findings suggest the concept of a strong metabolic component in the pathogenesis of OA [6,7], and highlight the association between DB and OA. Nevertheless, the mechanisms underlying the connection between both pathologies remain unclear.

Diabetes is a metabolic pathology with a high incidence and economic impact in terms of costs to society and health systems, so it is fast becoming the 21st century’s major public health concern. In this disease, impaired or scarce production of insulin contributes to an increase in the blood glucose levels, which is called hyperglycaemia. Hyperglycaemia-derived oxidative stress has been reported to initiate the development of DB complications [9,10], which is strongly correlated with the dysregulation of pro-inflammatory cytokine levels [10]. Likewise, similar oxidative stress disturbances can occur in OA [6,11]. Oxidative stress and related signalling pathways such as the activation of pro-inflammatory responses, participate in the changes that take place in the articular cartilage and predispose to OA onset [12,13]. Therefore, a growing number of studies have focused on the pro-oxidative actions that hyperglycaemia could activate in the OA joints from DB patients [14]. Elevated intracellular glucose levels mainly generate oxidative damage as a consequence of glycolytic pathway saturation in the cell, and the subsequent production of advanced glycation end products [9]. Nevertheless, impairment in the antioxidant defence system under hyperglycaemic conditions can also contribute to redox imbalance [9,10].

One of the mechanisms of anti-oxidant protection from glucotoxicity-induced oxidative stress is the production of hydrogen sulphide (H_2_S). H_2_S is a member of the gasotransmitter family, along with CO and NO, and as such, it can permeate cellular membranes without any specific transporter. In mammals, this gas is synthesised endogenously from l-cysteine by two pyridoxal-5′-phosphate-dependent enzymes, cystathionine β-synthase (CBS)m and cystathionine γ-lyase (CSE), and to a lower extent, by 3-mercaptopyruvate sulphurtransferase (MPST). It is currently accepted that both this gas and the necessary machinery for its synthesis are present in the joints [15]. H_2_S is involved in signalling in different systems, presenting a wide range of biological properties. In the joint, our group and others have reported that low concentrations of H_2_S exert anti-inflammatory and anti-oxidant effects in articular cells in OA models, both in vitro and in vivo [15,16,17,18]. It has also been described that H_2_S levels are reduced in DB, and that impaired H_2_S production could participate in its pathogenesis and associated complications [19,20]. Likewise, we have recently demonstrated that cartilage from OA patients presents a lower expression of CSE and, consequently, lower H_2_S levels than cartilage from non-OA patients [21].

Nonetheless, the role of H_2_S in DB has been controversial. There are numerous studies highlighting the pro-DB and toxic effects of H_2_S in the pancreas, and specifically in β-cells [22], which suggests that increased levels of pancreatic H_2_S promote DB development. However, it is now accepted that once DB is established and complications have arisen, there is a H_2_S deficiency at both systemic and peripheral levels [23,24]. It has also been shown that overexpression of CSE/CBS or treatment with H_2_S donors protects peripheral cells and tissues against the dysfunction triggered by high glucose or chemically-induced DB in both in vitro and in vivo models [25].

H_2_S is thought to act on several signalling pathways, mainly through a post-translational modification of proteins known as S-sulphydration [26]. Among these pathways, H_2_S promotes the activation of nuclear factor-erythroid 2-related factor-2 (Nrf-2), a master transcription factor involved in antioxidant signalling and the cell survival response, which regulates a wide battery of cytoprotective responses [23]. Recent studies have shown that Nrf-2 is a pivotal target for the prevention and attenuation of this disease [27] and for controlling bone and cartilage destruction induced by oxidative stress [28,29]. Under physiological conditions, Nrf-2 is generally located in the cytoplasm and is bound to its inhibitor, Kelch-like ECH-associated protein 1 (Keap1), which leads to its degradation. However, in response to oxidative stress or the presence of H_2_S, Nrf-2 dissociates from Keap1 after its sulphydration at Cys151 [30] and translocates to the nucleus to bind antioxidant-responsive elements in the promoter regions of its downstream antioxidant genes, including heme oxygenase-1 (HO-1) [31], which regulates metabolic processes in OA chondrocytes [29,32].

HO-1 overexpression in cartilage prevents the activation of catabolic, apoptotic, or senescence pathways induced by pro-inflammatory mediators [29,33]. Therefore, HO-1 represents an important part of the cell response to inflammatory and oxidative stress in joints. Interestingly, we have recently reported an impaired Nrf-2/HO-1 anti-oxidant axis in DB-related OA that could explain the greater inflammatory responsiveness of OA cartilage from this subset of patients [34]. However, the molecular mechanism responsible of this dysregulated Nrf-2/HO-1 expression has not been found yet. In this regard, the reduction in H_2_S production is now thought to be a potential mechanism to explain the impairment of this anti-oxidant axis in DB.

Taken together, these findings suggest that the impairment of H_2_S production observed in DB patients contributes to the devolvement of OA in this subset of individuals. To explore this hypothesis, we examined the modulation of H_2_S levels in serum and chondrocytes from OA patients with DB (DB-OA) or non-DB (non DB-OA) patients and in cells under glucose stress as well as its association with hyperglucidic-mediated dysregulation of chondrocyte catabolism. Furthermore, the involvement of impaired Nrf-2/HO-1 signalling was also studied.

## 2. Materials and Methods

The present study was reviewed and approved by the Research Ethics Committee of A Coruña-Ferrol, Spain. Samples were obtained from the Collection for Research on Rheumatic Diseases, authorised by the Galician Research Ethics Committee (2013/107) and inscribed in the Biobanks’ National Registry (C.0000424). All donors gave written informed consent. We employed three different set of patients for: (1) Measurement of serum H_2_S levels; (2) Analysis of H_2_S synthesizing enzymes in cartilage; and (3) Isolation and culture of human articular chondrocytes for in vitro experiments.

### 2.1. Measurement of H_2_S Levels in Serum Samples

An ion-selective microelectrode (model LIS-146GSCM; Lazar Research Lab. Inc., Los Angeles, CA, USA) attached to a voltage meter (Model 6230 N; Jenco Electronics, LTD, Taipei, Taiwan) was used for H_2_S quantification [21]. A calibration curve was prepared with Na_2_S standards (range from 1 × 10^4^ µM to 1 µM; 208043, Sigma-Aldrich Química S.A., Madrid, Spain). An anti-oxidant buffer stock solution was prepared with sodium salicylate, ascorbic acid, and NaOH in distilled water, according to the electrode instructions. This stock solution was further diluted 1:3 in distilled water to prepare the working solution. For H_2_S biosynthesis quantification in sera from patients, samples were mixed in polystyrene tubes with the same volume of the anti-oxidant buffer working solution. Tubes were sealed and incubated at 37 °C for 1 h. After this, the microelectrode was immersed in the fluid, and the voltage value was recorded and converted to H_2_S concentration with the calibration curve. Biosynthesis of H_2_S from cartilage was expressed as µM of H_2_S, mean ± SE. Values from OA individuals with and without DB were compared.

### 2.2. Isolation and Culture of Human Articular Chondrocytes

Samples of articular cartilage were collected from 12 OA patients (age 75.3 ± 13.3 years, six males and six females) who underwent orthopaedic surgery. Articular cartilage was sliced into small pieces and subjected to enzymatic digestion with 0.25% trypsin (Gibco, Thermo Fisher Scientific, Madrid, Spain) for 10 min and with 2 mg/mL type IV collagenase (Sigma-Aldrich Química S.A.) overnight at 37 °C. A 100 µm-pore filter was employed to filter the resulting cells, which were afterwards centrifuged at 430× *g* for 10 min, resuspended in Dulbecco’s modified Eagle’s medium (DMEM) with 4.5 g/L glucose (25 mM) (Lonza, Madrid, Spain), 10% foetal bovine serum (FBS, Gibco), and 1% penicillin/streptomycin (P/S, Gibco), and plated in adherent culture dishes (Costar Corning Incorporated, New York, NY, USA). Only freshly isolated chondrocytes or chondrocytes between the first- and the second-passages were used for the experiments. For silencing expression experiments, the human chondrocyte cell line TC28a2, widely used in in vitro models for studying molecular pathways involved in physiological and pathological processes in the cartilage [35], was employed.

### 2.3. Reagents and Treatment Conditions

Articular chondrocytes were seeded on 6-well plates (BD Biosciences, San Jose, CA, USA) for RNA and protein isolation; 12-well plates (BD Biosciences) for flow cytometry; and 96-well plates (BD Biosciences) for the IL-6 immunoenzymatic assay. After seeding, cells were cultured in DMEM with 1 g/L glucose (5.5 mM) (Gibco) and 10% FBS for at least one week. Then, quiescence was induced by culturing the cells in DMEM with 1 g/L glucose and 0.5% FBS for 48 h. Afterwards, experiments were performed in DMEM without FBS and with high glucose (4.5 g/L, HG) or low glucose (1 g/L, NG). IL-1β (5 ng/mL) (407615; Sigma-Aldrich Química S.A.) was employed to induce an inflammatory response, and the effect of the H_2_S donors NaSH (500 µM) (161527; Sigma-Aldrich) and GYY-4137 (500 µM) (sc-224013; SantaCruz Biotechnology, Heidelberg, Germany) in these two different environments (HG vs. NG) was evaluated.

### 2.4. Protein Isolation, SDS-PAGE, and Western Blot

After stimulation with IL-1β and H_2_S donors for 24 h, cultured chondrocytes were washed with cold saline buffer (Fresenius Kabi, Barcelona, Spain) and total protein was extracted employing Tris-HCl buffer pH 7.5 containing inhibitor cocktail and 1% phenylmethylsulphonyl fluoride (all from Sigma-Aldrich). Protein from freshly isolated chondrocytes was extracted, quantified in a ND-1000 UV–Vis Spectrophotometer (NanoDrop Technologies LLC, Thermo Fisher Scientific), and was used to determine protein concentration. Afterwards, proteins were separated by sodium dodecyl sulphate–polyacrylamide gel electrophoresis (SDS-PAGE) as previously described [34]. Once separated, proteins were transferred to polyvinylidene difluoride membranes (Millipore, Burlington, MA, USA) in a Trans-Blot SD Semi-Dry Transfer Cell (Bio-Rad Laboratories S.A., Madrid, Spain). Membranes were then incubated with anti-human CBS (1:200; ab54883) and CSE (1:200; ab54573) (Abcam, Cambridge, UK), MPST (1:200; sc-376168), Nrf-2 (1:1000; sc-13032) (SantaCruz Biotechnology), HO-1 (1:1000; ADI-SPA-895; Enzo Life Sciences, New York, USA), COX-2 (1:250; MAB4198; R&D Systems), and tubulin (1.3000; T9026; Sigma-Aldrich) antibodies overnight at 4 °C. After washing, membranes were incubated with the anti-secondary antibody (NA931 and NA934 for anti-mouse and anti-rabbit IgG, respectively; VWR International, Lutterworth, UK) and ECL chemiluminescent substrate (Millipore) was used for detecting antigen-antibody binding. Protein bands were quantified by densitometry with the ImageQ image processing software (http://imagej.nih.gov/; 30 December 2021). All protein band intensities were normalized to the tubulin band intensity for the same sample.

### 2.5. RNA Isolation, RT-PCR, and qPCR

After 24 h of stimulation with IL-1β and H_2_S donors, RNA was extracted and purified using the TRIzol Reagent (Invitrogen, Thermo Fisher Scientific, Waltham, MA, USA), chloroform, and isopropanol (Sigma-Aldrich Química, S.A.), and quantified with a ND-1000 UV–Vis spectrophotometer. Reverse transcription (RT-PCR) of 500 ng of RNA from each sample was performed with the NZY First-Strand cDNA Synthesis Kit (Nzytech, Lisboa, Portugal) in a 96-well Thermal Cycler (Applied Biosystems, Thermo Fisher Scientific). Quantitative real-time polymerase chain reaction (qPCR) experiments were run on a LightCycler 480 instrument, employing LightCycler 480 SYBR Green I Master (Roche Diagnostics, Sigma-Aldrich Química S.A.) and gene-specific primers purchased from Invitrogen (shown in Table 1). Ribosomal protein large P13 (RPLP13) for primary cultured chondrocytes and tyrosine 3-monooxygenase/tryptophan 5-monooxygenase activation protein zeta (YWHAZ) for T/C28a2 cells were employed as the reference gene for normalisation. Data was analysed with the LC480 software, version 1.5 (Roche Diagnostics), and relative expression levels were calculated with the 2^−ΔΔCT^ method.

### 2.6. Flow Cytometric Analysis of ROS Production

The production of reactive oxygen species (ROS) by chondrocytes was analysed after 24-h stimulation with IL-1β and H_2_S donors. Briefly, cells were split with 0.1% trypsin-EDTA (Gibco), washed twice in PBS, and incubated with 2′,7′-diclorodihidrofluorescein (DCFDA) (D399; Thermo Fisher Scientific). After incubation, cells were washed, resuspended in PBS, and transferred to polypropylene tubes (NUNC, VWR International). Data acquisition was made using a BD FACSCalibur flow cytometer (BD Biosciences, Madrid, Spain), and the data obtained were analysed using BD Cell-Quest Pro software (BD Biosciences). For each assay, a minimum of 10^5^ cell events were acquired. Results are shown as percentage of positive cells.

### 2.7. Immunoenzymatic Assay of IL-6 Production

The levels of interleukin 6 (IL-6) in culture supernatants from articular chondrocytes after 48 h treatment with IL-1β and H_2_S donors were determined with the DuoSet ELISA Kit for human IL-6 (DY206; Bio-Techne R&D Systems, Madrid, Spain), following the manufacturers’ instructions. Data were expressed as released picograms per mL The working range was between 9.38 and 600 pg/mL.

### 2.8. Nrf-2 Silencing

T/C28a2 cells were seeded on 12-well plates and cultured in DMEM and 10% FBS for one week. Then, cells were transfected with three different Nrf-2 siRNA or non-coding siRNA as the control at a final concentration of 300 nM (Ambion, ThermoFisher) and 25 µL (5% *v*/*v*) of X-tremeGENE HP DNA Transfection Reagent (6366236001; Roche Diagnostics) in DMEM with NG and 0.5% FBS. This mixture was incubated for 25 min at room temperature and added dropwise to culture dishes. After 24 h, transfected cells were cultured for 24 h in DMEM with high levels of glucose in the presence or absence of IL-1β (5 ng/mL) and the H_2_S donors NaSH (500 µM) and GYY-4137 (500 µM). Afterwards, RNA and proteins were isolated and IL-6 levels in culture supernatants were analysed as previously described.

### 2.9. Immunohistochemistry

Immunohistochemical studies were performed in sections of paraffin-embedded joints from lean and db/db mice (mice with mutated receptor of leptin) obtained from a previous study of our group [36]. Samples were deparaffinised, cleared with xylene, and hydrated in a series of increasing grade alcohol. Heat-mediated antigen retrieval was performed in citrate buffer (pH 6.0; S2369; Dako, Agilent Technologies Spain S.L., Barcelona, Spain) for Nrf-2 detection or in ethylenediaminetetra-acetic acid (EDTA) buffer (pH 9.0; S2367; Dako) for CBS and CSE detection. Thereafter, peroxidase blocking solution (Dako) was used to block endogenous peroxidase activity. Then, slides were washed with phosphate buffer solution and incubated with primary antibodies against Nrf-2 (1:200; sc-13032; Santa Cruz Biotechnology), CBS (1:400; ab54883; Abcam), and CSE (1:400; ab54573; Abcam). Antigen–antibody interactions were determined with the rabbit/mouse peroxidase/DAB DAKO REAL EnVision Detection Kit (K5007; Dako). Sections were counterstained with haematoxylin and eosin. Slides were dehydrated in graded alcohol, cleared in xylene, and mounted in DePeX (Dako). Slides were visualised in an Olympus Dx61 optical microscope (Olympus España S.A.U., Barcelona, Spain). Staining intensity was quantified using ImageJ software.

### 2.10. Statistical Analysis

All data in the graphs are reported as points representing one sample or a single experiment obtained from one single patient, with standard error of the mean (SEM) to represent error. Means of the variables tested from “n” independent samples or experiments (n = number of patients) are also shown in graphs. Unsupervised hierarchical cluster analysis (HCA) was carried out in MetaboAnalyst (McGill University, Sainte Anne de Bellevue, QC, Canada) based on Pearson correlation distance and average cluster algorithm. Data used for HCA analysis were log transformed using the base-2 logarithm (log 2) to correct for non-normal distributions and standardised (mean centred divided by standard deviation). The GraphPad PRISM version 5 statistical software package (La Jolla, CA, USA) was used to perform statistical analysis. The nonparametric Mann–Whitney test and Wilcoxon test were used to compare different patients and treatments. Statistically significant differences between experimental conditions were determined by the paired comparison test. A difference was considered significant with *p*-value ≤ 0.05.

## 3. Results

### 3.1. Reduced Levels of Serum H_2_S in OA Patients with DB Are Associated with Hyperglycaemia

**Figure 1:**
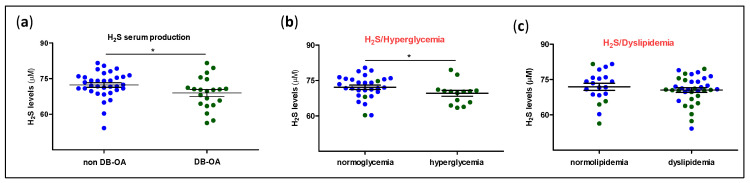
Serum H_2_S levels in osteoarthritic patients with or without diabetes. Using an ion-selective microelectrode, (**a**) the levels of H_2_S were measured in the sera from osteoarthritis patients without diabetes (non DB-OA, blue dots; n = 34) or with diabetes (DB-OA, green dots; n = 21). Additionally, the study population was divided into patients with normoglycaemia (n = 29) or hyperglycaemia (n = 14) (**b**) or individuals with normolipidaemia (n = 20) or dyslipidaemia (n = 35) background (**c**) and H_2_S levels were analysed in these subsets of patients. Values are mean ± SEM. * *p* ≤ 0.05 analysed by the Mann–Whitney test.

To determine the existence of an impaired H_2_S production at systemic level in a DB subset of OA patients, serum samples were obtained from OA individuals with or without DB, and the levels of H_2_S were measured by an ion-selective microelectrode. As shown in Figure 1a, OA-DB patients presented a significantly lower production of this physiological gas than those from non-DB donors. Socio-demographic and clinical characteristics of our study population were also analysed (Table 2). As previously matched, no differences were observed between both subset of patients in terms of sex, age, and BMI. However, as expected, DB individuals showed higher levels of glucose and hence more incidence of hyperglycaemia than in non-DB patients. Furthermore, we also detected differences between both sets of patients when history of dyslipidaemia was evaluated, with this pathology also having significantly higher incidence in DB-OA donors. In order to analyse the influence of hyperglycaemia and dyslipaemia on H_2_S production, we sorted the study patients depending on the presence or absence of these clinical features and independently on DB diagnosis. As shown in Figure 1b,c, patients with a dyslipidaemia background did not show different values of H_2_S from those with normolipidaemia, whereas glycaemia was found to influence serum levels of this gas, which were lower in those donors with hyperglycaemia.

### 3.2. Expression of H_2_S Synthesizing Enzymes Is Decreased in the Cartilage from DB-OA Patients and Associated with Hyperglycaemia

**Figure 2:**
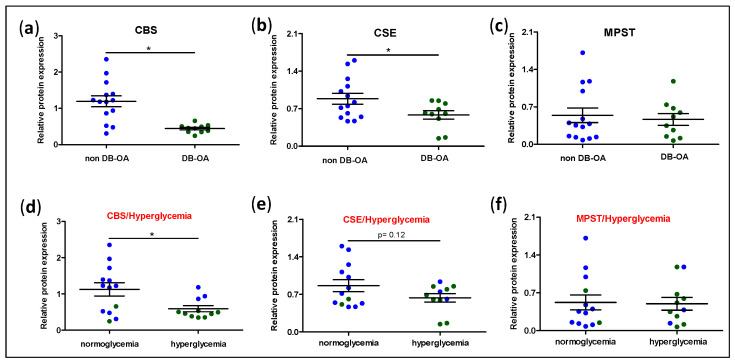
Levels of H_2_S synthesising enzymes in cartilage from osteoarthritic patients with or without diabetes. The protein expression of enzymes involved in H_2_S biosynthesis, (**a**) cystathionine β-synthase (CBS), (**b**) cystathionine γ-lyase (CSE), and (**c**) 3-mercaptopyruvate sulphurtransferase (MPST), was evaluated by western blot in freshly isolated chondrocytes from OA patients without DB (non DB-OA, blue dots; n = 14) or with DB (DB-OA, green dots; n = 10). Additionally, the study population was divided into patients with normoglycaemia or hyperglycaemia and (**d**–**f**) levels of these enzymes were analysed in these different subsets of patients. Values were mean ± SEM. * *p* ≤ 0.05 analysed by the Mann–Whitney test.

To measure the machinery of H_2_S biosynthesis at the local level in the joint, we analysed the expression of CBS, CSE, and MPST, the main enzymes involved in H_2_S synthesis, in cartilage samples from OA patients with or without DB. Interestingly, protein expression of CBS and CSE were downregulated in the cartilage of OA-DB donors, whereas MPST levels were not modulated (Figure 2a–c). As we employed a different group of patients from that used to study the serum levels of H_2_S, socio-demographic and clinical characteristics of this new study population were also analysed (Table 3). No differences regarding sex, age, BMI, history of dyslipidaemia or blood levels of cholesterol and triglycerides were observed between OA individuals with and without DB. In contrast, higher levels of glucose and incidence of hyperglycaemia were detected in the subset of patients with DB. Interestingly, when the study population was divided into the normoglycemic and hyperglycaemic groups and the expression of H_2_S synthesising enzymes were analysed, we observed that both CBS and CSE, but not MPST, were downregulated in the hyperglycaemic group, with these differences only statistically significant for CBS expression (Figure 2d–f).

Additionally, we investigated whether differences in the medication received by the DB individuals compared with non-DB ones could also explain the modulation of H_2_S production or the expression of H_2_S synthesising enzymes detected in these subsets of patients. As shown in Table 4, few differences regarding pharmacologic treatment were found between DB and non-DB individuals. We only detected a significantly higher consumption of drugs for hypercholesterolemia and hypertension and anti-aggregation/coagulation in DB donors of sera and cartilage, respectively. However, after a comparison between the treated and non-treated patients, we did not observe any influence of these treatments on the modulation of systemic H_2_S levels or cartilage expression of H_2_S synthesising enzymes.

### 3.3. Expression of HO-1 Is Decreased in Cartilage from DB-OA Patients and Associated with Hyperglycaemia and CBS Levels

**Figure 3:**
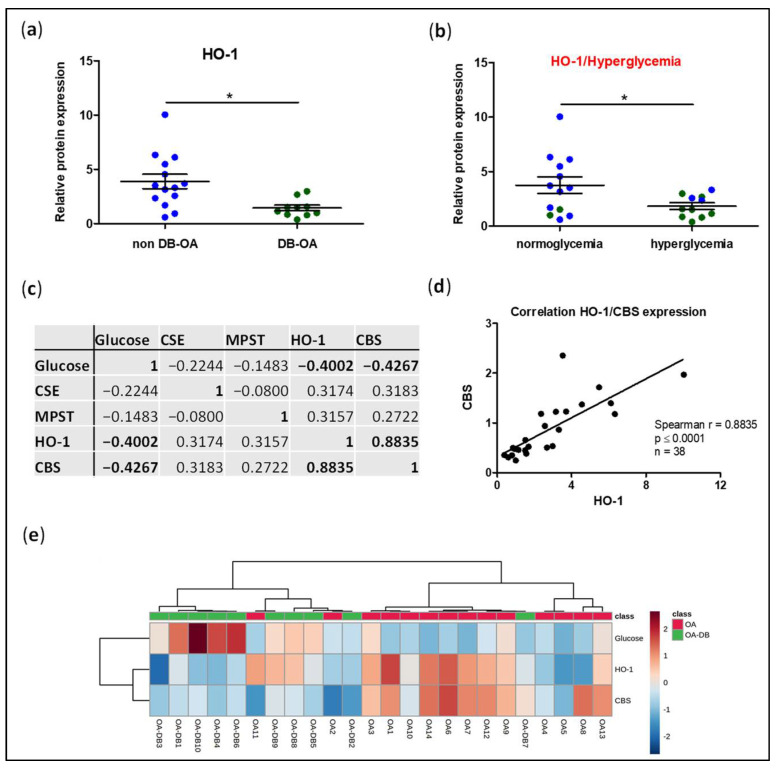
HO-1 expression in cartilage from osteoarthritic patients with or without diabetes and its association with H_2_S synthesising enzymes. (**a**) The protein expression HO-1 was evaluated by western blot in freshly isolated chondrocytes from OA patients without DB (non DB-OA, blue dots; n = 14) or with DB (DB-OA, green dots; n = 10). Additionally, samples were grouped into patients with normoglycaemia or hyperglycaemia (**b**) and levels of HO-1 were analysed in these different subsets of individuals. (**c**) Association between glucose levels, HO-1 and H_2_S synthesising enzymes (*p* ≤ 0.05 shown in bold). (**d**) Graph representing Spearman correlation between HO-1 and CBS. Additionally, linear regression is shown. (**e**) Hierarchical clustering heatmap analysis was carried out to identify different OA phenotypes based on a specific profile of variables. Values are mean ± SEM. * *p* ≤ 0.05 analysed by the Mann–Whitney test. CBS, cystathionine β-synthase; CSE, cystathionine γ-lyase; HO-1, hemeoxygenase-1; MPST, 3-mercaptopyruvate sulphurtransferase.

In a previous work, we identified an impairment in Nrf-2/HO-1 protein expression in the cartilage from DB-OA patients [34]. To further confirm these findings, we evaluated whether protein levels of HO-1 were also reduced in our study population and whether its expression was associated with hyperglycaemia. As expected, fresh isolated chondrocytes from the cartilage of DB-OA patients showed lower levels of HO-1 than those from non DB-OA patients (Figure 3a). Furthermore, we observed a significant reduction in HO-1 expression in those patients with hyperglycaemia (Figure 3b). Since H_2_S is an inductor of the Nrf-2/HO-1 antioxidant pathway, we next analysed a putative association between impairment of HO-1 expression and reduced levels of H_2_S synthesising enzymes in the cartilage. As shown in Figure 3c,d, HO-1 showed a strong and positive correlation with CBS expression. Moreover, we also observed a negative association between glucose levels and both HO-1 and CBS expression. However, we failed to detect any significant correlation between CSE/MPST expression and glucose or HO-1 levels. Likewise, we performed a hierarchical clustering heatmap in order to investigate whether different OA phenotypes based on a specific profile of altered variables could be defined (Figure 3e). Interestingly, analysis revealed two clusters of patients based on their pattern of expression in HO-1, CBS, and serum levels of glucose, which generally coincided with belonging to the DB or non-DB subgroup of patients.

### 3.4. HG Exposition Attenuates the Expression of H_2_S Synthesizing Enzymes in IL-1β-Activated Chondrocytes. H_2_S Donors Modulate IL-1β-Induced Pro-Catabolic Response in Chondrocytes under HG Environment

**Figure 4:**
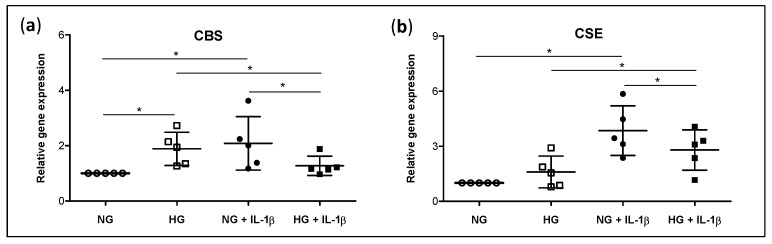
IL-1β-stimulated expression of H_2_S synthesising enzymes under high glucose environment. OA human chondrocytes were incubated in growth media with normal (NG) or high (HG) levels of glucose in the presence or absence of IL-1β. Thereafter, the gene expression of (**a**) CBS and (**b**) CSE was analysed. Values are mean ± SEM (n = 5). * *p* ≤ 0.05 analysed by the Wilcoxon test. CBS, cystathionine β-synthase; CSE, cystathionine γ-lyase.

To further confirm the impact of glucose stress in the previously described findings, we performed an in vitro model that mimicked the DB-OA environment in the cartilage by exposing human cultured chondrocytes to HG stress in the presence of IL-1β, one of the key pro-catabolic cytokines involved in OA pathogenesis. We first evaluated the gene expression of CBS and CSE, the main enzymes involved in H_2_S synthesis that were found dysregulated in our previous analysis. The incubation of chondrocytes in HG upregulated the expression of CBS, reaching significant differences versus NG condition (Figure 4a). Similarly, IL-1β clearly increased the expression of CBS and CSE in those cells incubated in normal levels of glucose. However, the IL-1β-elicited expression of both enzymes was significantly attenuated when chondrocytes were cultured in a HG environment (NG + IL-1β vs. HG + IL-1β) (Figure 4a,b). In addition, the co-treatment of cells with HG + IL-1β reverted the increment of CBS expression induced by HG alone, whereas it enhanced the CSE levels (HG vs. HG + IL-1β) (Figure 4a,b).

**Figure 5:**
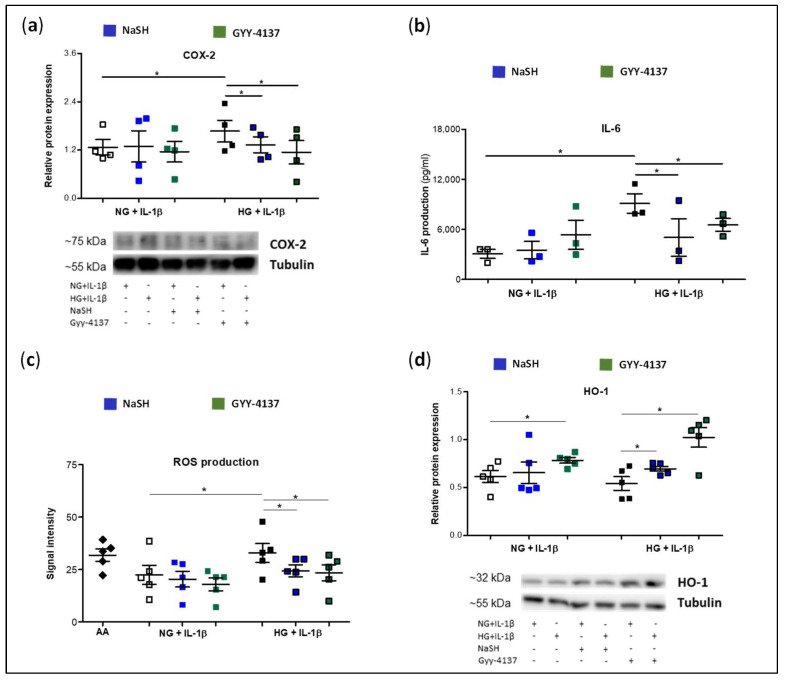
Effect of exogenous administration of H_2_S on pro-inflammatory and pro-oxidant responses to IL-1β and HO-1 expression recovery in chondrocytes exposed to a HG environment. OA human chondrocytes were incubated in growth media with normal (NG) or high (HG) levels of glucose in the presence or absence of IL-1β and co-treated with a fast or slow-releasing H_2_S donor, NaSH 500 µM and GYY-4137 500 µM, respectively. Then, (**a**) protein levels of COX-2 were measured by western blot. Below panel shows representative images of COX-2 expression from one experiment. Expression levels of β-tubulin were employed as the loading control. (**b**) IL-6 released in the cell culture supernatant was measured by ELISA. (**c**) ROS production was monitored by flow cytometry using the fluorogenic dye 2′, dichlorofluorescein (DCF). Antimycin A (AA) was employed as a positive control of ROS production. (**d**) Protein HO-1 expression was also evaluated. Below panel shows representative images of HO-1 expression from one experiment. Values are mean ± SEM. * *p* ≤ 0.05 analysed by the Wilcoxon test. COX-2, cyclooxygenase 2; HO-1, hemeoxygenase-1; IL-6, interleukin 6.

In a previous work, we detected that HG exposition exacerbated the inflammatory and oxidative response induced by IL-1β in murine chondrocytes [34]. Thus, to evaluate the involvement of H_2_S biosynthesis impairment in this situation, human chondrocytes were co-treated with a fast- or slow-releasing H_2_S donor, NaSH and GYY-4137, respectively. Thereafter, the effect of boosting H_2_S levels under the different experimental conditions was analysed. As shown in Figure 5a–c, IL-1β elicited a significant higher COX-2 protein expression, IL-6 release, and ROS production in those cells incubated in HG than in those cultured in NG, confirming our previous findings. Treatment with H_2_S donors attenuated this effect, reducing the levels of pro-inflammatory and pro-oxidative mediators induced by IL-1β in HG conditions. Conversely, we observed an inconsistent impact of H_2_S donors on pro-catabolic responses stimulated by IL-1β in chondrocytes incubated in NG (Figure 5a–c). Additionally, we evaluated whether upregulation of protein HO-1 levels underlies the beneficial effect of H_2_S-releasing molecules. In accordance with the previous findings, treatment of chondrocytes with NaSH and GYY-4137 significantly recovered the reduced expression of HO-1 triggered by IL-1β under HG conditions, whereas scarce effects were observed in those cells incubated in NG, where only GYY-4137 treatment showed a significant but modest increment in the HO-1 values under IL-1β stimulation (Figure 5d).

### 3.5. Nrf-2/HO-1 Pathway Is Involved in the Modulation of H_2_S Biosynthesis and in the Anti-Inflammatory Effects of Exogenous Administration of H_2_S under HG Environment

**Figure 6:**
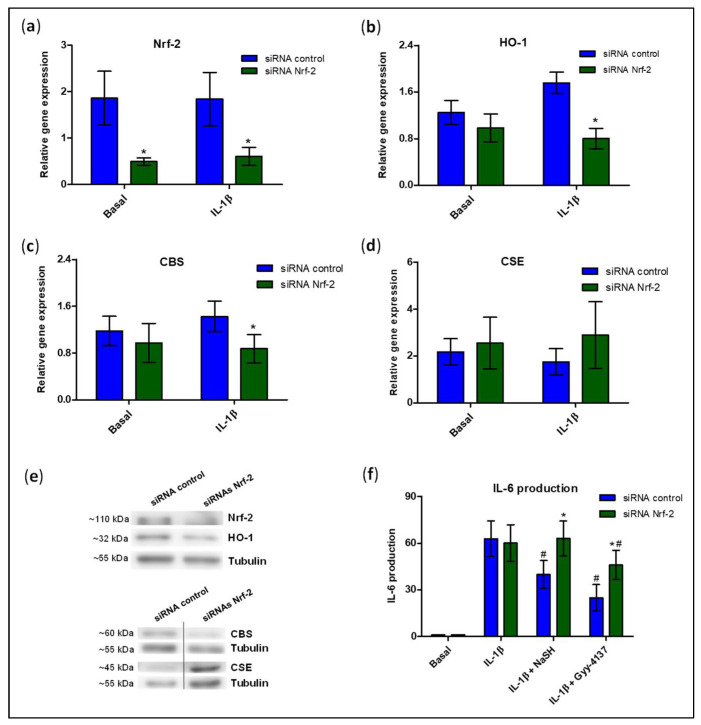
siRNA-induced reduction in Nrf-2 expression modulates HO-1 and CBS levels and attenuates anti-inflammatory effects of exogenous administration of H_2_S on IL-1β-stimulated chondrocytes under a HG environment. TC28a2 cells were transfected with the control or NRF-2 siRNA and then incubated in growth media with high levels of glucose in the presence or absence of IL-1β. Then, the gene expression of (**a**) Nrf-2, (**b**) HO-1, (**c**) CBS, and (**d**) CSE were analysed. (**e**) Representative images of one experiment evaluating protein expression of Nrf-2, HO-1, CBS, and CSE by western blot. Protein levels of tubulin were used as the loading control. (**f**) IL-6 production was analysed by ELISA. Basal condition in cells transfected with siRNA = 1. Values are mean ± SEM. * *p* ≤ 0.05 vs. respective condition in cells transfected with siRNA control, analysed by the Wilcoxon test. ^#^ *p* ≤ 0.05 vs. cells treated with IL-1β alone (n = 3). CBS, cystathionine β-synthase; CSE, cystathionine γ-lyase; HO-1, hemeoxygenase-1; IL-6, interleukin 6; Nrf-2, nuclear factor erythroid 2-related factor 2.

Considering the results shown above, together with our previous observations that Nrf-2/HO-1 signalling is defective in cartilage from OA-DB patients [34], we evaluated whether a reduction in Nrf-2 levels could affect the expression of H_2_S synthesising enzymes. The gene expression of Nrf-2 was partially blocked by siRNA in the human chondrocyte cell line TC28a2. As shown in Figure 6a, the gene levels of Nrf-2 were significantly reduced by approximately 70%, and later confirmed at the protein level (Figure 6e). Additionally, HO-1 gene expression was attenuated in those cells transfected with Nrf-2 siRNA, achieving significant differences in the chondrocytes stimulated with IL-1β (Figure 6b)). Thereafter, we measured the gene expression of CBS and CSE, observing a reduction in the levels of CBS in the IL-1β-treated cells with Nrf-2 expression partially blocked (Figure 6c), whereas we failed to observe any significant modulation of CSE levels (Figure 6d). These modulations were additionally observed at the protein level (Figure 6e).

Finally, we further explored the possibility that the beneficial effects of exogenously boosting H_2_S levels in chondrocytes were mediated through activation of Nrf-2/HO-1 signalling. Therefore, IL-6 production was assayed in chondrocytes transfected with Nrf-2 or control siRNA and subsequently treated with H_2_S donors. As expected, NaSH and GYY-4137 significantly attenuated IL-1β-induced production of IL-6 in the control siRNA transfected cells (Figure 6f). Conversely, the anti-inflammatory actions of H_2_S donors were significantly attenuated in those chondrocytes with reduced Nrf-2 expression (transfected with Nrf-2 siRNA), even showing a complete loss of NaSH treatment effect.

### 3.6. Reduced H_2_S Biosynthesis and Nrf-2 Expression in db/db Mice Is Associated with a Higher Predisposition to Cartilage Damage in a Surgically Induced OA Model

**Figure 7:**
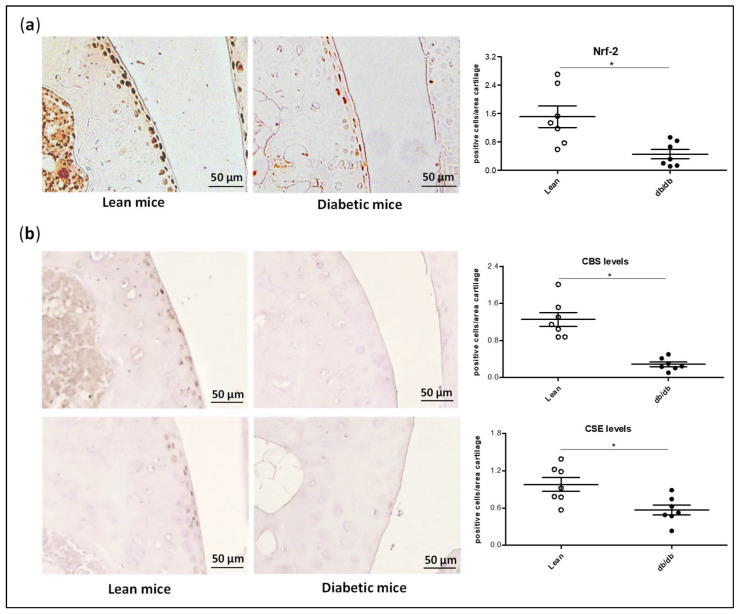
Expression of Nrf-2 and H_2_S synthesising enzymes is diminished in the cartilage from diabetic mice. Histological slides of joints from lean or diabetic (db/db) mice were employed to analyse cartilage expression of (**a**) Nrf-2 and (**b**) H_2_S synthesising enzymes CBS and CSE by immunohistochemistry. Images on the left show representative micrographs from one experiment (n = 7). Values are mean ± SEM. * *p* ≤ 0.05 CBS, cystathionine β-synthase; CSE, cystathionine γ-lyase; Nrf-2, nuclear factor erythroid 2-related factor 2.

In a previous study by our group, we detected that db/db mice (animals with mutated receptor of leptin) showed higher levels of blood glucose and insulin than lean mice, and presented aggravated cartilage damage after experimental OA [36]. Thus, to further confirm the involvement of an impairment of Nrf-2/H_2_S pathways in OA pathogenesis in DB patients, we measured the expression of Nrf-2 and the main H_2_S synthesising enzymes in the cartilage from lean and db/db mice. We observed that db/db mice had lower levels of Nrf-2 than lean mice, as shown by immunohistochemistry (Figure 7a). Similarly, CBS and CSE expression was also significantly reduced in the cartilage from these animals (Figure 7b). Interestingly, we also detected a significant correlation between Nrf-2 expression and CBS and CSE expression (Spearman r = 0.55 and 0.57, respectively; *p* < 0.05), and between CBS and CSE expression (Spearman r = 0.64; *p* < 0.05).

## 4. Discussion

The role of H_2_S in the development and pathogenesis of DB and its associated complications has been the source of debate in the recent years. Nonetheless, it is now widely accepted that an increment of H_2_S in the pancreas and liver at the early stages of the disease could promote insulinemia and hyperglycaemia, and in turn, participate in DB pathogenesis. Conversely, when the disease is already established, an impairment in H_2_S production at the peripherical tissue level can favour the activation of pathological pathways, triggering DB-related disorders [23,24,37].

Likewise, metabolic phenotype of OA is now considered to be another complication associated with DB [7,8]. However, the underlying mechanisms linking both diseases are still elusive. In an attempt to further elucidate this question, in this study, we observed, for first time to our knowledge, the existence of a decline in H_2_S biosynthesis in cartilage from OA patients with DB. Interestingly, the reduced production of H_2_S at local and systemic levels detected in this subset of OA patients was clearly associated with hyperglycaemia, and seems to be responsible for the chondrocyte dysfunction that contributes to OA pathogenesis. This dysfunction includes exacerbated inflammatory and oxidative responses as we have observed in an in vitro model of high glucose-induced stress in IL-1β-activated chondrocytes. Furthermore, exogenous administration of H_2_S recovers the regulation of chondrocyte catabolism through the activation of Nrf-2/HO-1 pathways, suggesting H_2_S induction as a promising strategy in the treatment of DB-related OA.

Different studies have demonstrated that the reduction in serum H_2_S levels observed in DB patients is associated with hyperglycaemia or a worst control of glucose levels [20,38]. In addition, the incidence of complications associated with this pathology such as cardiovascular disorders is higher in those patients with lower serum levels of H_2_S, suggesting the involvement of H_2_S in the pathogenesis of DB-related conditions [20]. Accordingly, we detected that OA patients with DB showed lower serum H_2_S levels than individuals without DB. Furthermore, we identified that hyperglycaemia, rather than other social-demographical or clinical characteristics that differed between both sets of patients, was associated with reduced H_2_S values. Nevertheless, previous works have observed that adiposity or BMI can also affect the systemic level of this gas [38]. In our study, these critical variables were matched to discard their influence on the levels of H_2_S. Nonetheless, more studies will be needed to clarify the influence of other metabolic parameters in the modulation of serum H_2_S values.

Local impairment of H_2_S biosynthesis is now considered as a key event in the pathogenesis of DB-related complications. For instance, different studies have described that reduced expression of CSE or/and CBS favours the development/progression of diabetic retinopathy [39], cardiopathy [40,41,42], and nephropathy [43,44]. Accordingly, in our study, chondrocytes from the cartilage of DB-OA patients presented a reduced expression of CBS and CSE, although we failed to detect any modulation of MPST, the H_2_S-synthesising enzyme mainly located in the mitochondria [45]. However, we and other authors have previously detected a lower expression of MPST, together with a slight reduction in H_2_S levels in the cartilage of OA patients in comparison with healthy donors [21,46]. This phenomenon could be related to the fact that chondrocytes from OA patients present dysfunctional mitochondria [1,47]. We speculate that mitochondrial dysfunction in OA may be associated with an alteration in MPST levels, as different findings suggest [48,49]. Therefore, the fact that the current study was carried out only in samples from OA patients could account for the lack of modulation of MPST. Nevertheless, future studies will be warranted to further elucidate these observations.

DB is associated with a number of comorbidities and risk factors such as atherosclerosis, hypertension, or dyslipidaemia, among others [50,51]. Thus, DB patients are more likely to be under different drug therapies than non-DB individuals. As expected, we detected a higher percentage of DB than non-DB patients having treatment against atherosclerosis, hypercholesterolemia, and aggregation/coagulation dysregulation in our study population. Since there are strong indications that some of these conditions as well as their treatments could modulate H_2_S levels [42], we discarded the influence of these treatments on H_2_S levels and its biosynthesis machinery. Conversely, we further confirmed a strong correlation between CBS expression and hyperglycaemia. In this regard, hyperhomocysteinemia, a condition caused by the lack of CBS expression and characterised by elevated levels of plasma homocysteine, has been associated with DB pathogenesis [52]. This fact, along with the synergistic relationship between hyperglycaemia and hyperhomocysteinemia [53,54], could explain the negative CBS/hyperglycaemia correlation detected in our study. Additionally, recent studies have observed that hyperhomocysteinemia was related to a reduction in Nrf-2/HO-1 expression [55,56], a finding in accordance with the lower levels of CBS and Nrf-2 detected in OA-DB patients and db/db mice in our current and previous studies [34].

To further elucidate the association between hyperglycaemia and H_2_S biosynthesis in DB-OA cartilage, we performed an in vitro model of glucose-induced stress in IL-1β-activated chondrocytes. Incubation of chondrocytes with high levels of glucose downregulated IL-1β-induced expression of CBS and CSE, the main enzymes involved in H_2_S biosynthesis, mimicking what we previously observed in the cartilage from DB-OA patients. Accordingly, previous in vitro studies have also shown that different cell types under high glucose stress reduce the expression of CBS [57,58] and CSE [59,60]. Furthermore, as previously detected [34], the incubation of chondrocytes with high glucose also exacerbated the pro-inflammatory and oxidative response to IL-1β. Interestingly, the addition of exogenous sources of H_2_S attenuated these effects, but failed to modulate the response to IL-1β in cells under normal levels of glucose. Similarly, different authors have observed that chemical upregulation of H_2_S levels protects against high glucose-elicited pathological responses both in in vitro and in vivo experimental approaches of DB or its associated complications [57,59,60,61]. These findings suggest that the key role of H_2_S biosynthesis impairment on the disruption of cartilage homeostasis, which subsequently favours the activation of pathological pathways in the chondrocytes from DB-OA patients.

H_2_S plays a pivotal role in several physiological pathways such as those involved in the regulation of metabolic and redox homeostasis or in the control of the inflammatory response [23,37]. Thereby, reduced levels of H_2_S could compromise these pathways. An important number of H_2_S actions are mediated through the activation of Nrf-2 signalling pathways and upregulation of gene transcriptional targets including HO-1 [23,30]. Interestingly, in a previous work, we detected that the Nrf-2/HO-1 antioxidant axis was defective in the cartilage from DB-OA patients, and that this defect was related to their greater inflammatory responsiveness [34]. In the current study, we further confirmed the decline in HO-1 expression in DB-OA cartilage, and we observed that induction of H_2_S production reverted the downregulation of HO-1 levels induced by high glucose stress in vitro. To determine whether the H_2_S effects on cartilage homeostasis were mediated by the regulation of the Nrf-2/HO-1 signalling pathway, as our findings have suggested, we partially blocked Nrf-2 expression in a chondrocyte cell line. As expected, the reduction in Nrf-2 levels lowered HO-1 protein levels, since it is widely known that this transcriptional factor is the most important regulator of HO-1 expression [29,62]. Interestingly, CBS expression was also attenuated in those chondrocytes with Nrf-2 expression partially blocked. Accordingly, Liu et al. (2019) identified a putative ARE in the CBS promoter region that could be responsible for Nrf-2-activated CBS promoter activity, and detected that inhibition of Nrf-2 expression caused CBS downregulation [63]. A reduction in CBS levels could trigger an accumulation of its enzymatic substrate, homocysteine, which has been described to upregulate CSE expression [40]. This could explain that chondrocytes with reduced Nrf-2 expression failed to downregulate CSE levels or even increased its expression in our experiments. We also observed that the blockage of Nrf-2 expression significantly mitigated the anti-inflammatory effects of exogenous H_2_S addition. Similarly, a growing number of studies have detected that H_2_S upregulation protected cells from high glucose- or DB-induced catabolism through activation of Nrf-2 pathways [30,64,65]. It has also been described that Nrf-2 deficiency partially abrogates H_2_S-mediated inhibition of oxidative stress and pro-inflammatory responses [30,34,66,67].

Finally, our findings were further confirmed in db/db mice, a well-established model of DB with hyperglycaemia and hyperinsulinemia [36]. The expression of Nrf-2, CBS, and CSE was significantly reduced in the cartilage from mice with the mutated leptin receptor compared to wild type mice. Interestingly, in a previous study from our group, we observed that db/db mice also showed increased joint damage after experimental OA [36], suggesting an association between both events. Other studies have also described a reduction in Nrf-2 levels in peripheral tissues and organs from db/db mice [68,69], although no research had examined Nrf-2 expression at the cartilage level in these mice to date. Likewise, impairment of H_2_S and the enzymes involved in its biosynthesis has been detected in numerous studies using db/db mice as well as in other in vivo models of diabetes [64,70,71,72]. Additionally, these studies detected that the addition of exogenous sources of H_2_S protected against different pathologies observed in diabetic mice [64,70,71]. All together, these findings reinforce the hypothesis that a decline in the Nrf-2/H_2_S axis in DB is a central event involved in its pathogenesis and is related to its associated complications including OA.

## 5. Conclusions

In this study, we have observed, for the first time to our knowledge, that DB-OA patients showed a reduction in serum H_2_S levels as well as a decline in H_2_S biosynthetic machinery at the articular cartilage level. These observations are strongly associated with our previous findings in detecting an impairment of the Nrf-2/HO-1 signalling pathway and higher inflammatory responsiveness in chondrocytes from OA-DB individuals [34]. Our results indicate that H_2_S is an inductor of the Nrf-2/HO-1 signalling pathway in cartilage, but also a downstream target of the transcriptional activity of Nrf-2. An impairment of H_2_S and/or Nrf-2 levels under glucose stress or DB triggers chondrocyte catabolic responses, favouring the disruption of cartilage homeostasis that characterises OA pathology. Additionally, based on the protein expression and biochemical profile of our study population, we were able to identify two distinctive groups of OA patients that nearly corresponded with those from the DB and non-DB subsets. This fact is of special interest as OA is a phenotypically heterogeneous disease, and thus the development of reliable tools or diagnostic markers for the stratification of patients will be pivotal to implement personalised medicine. Finally, our findings highlight the benefits of the use of exogeneous sources of H_2_S in the treatment of OA patients with DB, and warrant future clinical studies.

## Figures and Tables

**Table 1:** Sequence of primers used to analyse gene expression. CBS, cystathionine β-synthase; CSE, cystathionine γ-lyase; HO-1, heme oxygenase-1; MPST, 3-mercaptopyruvate sulphurtransferase; Nrf-2, nuclear factor-erythroid 2-related factor-2; RPL13, ribosomal protein large P13; YWHAZ, tyrosine 3-monooxygenase/tryptophan 5-monooxygenase activation protein zeta.

	Reference Sequence	Forward Sequence	Reverse Sequence
CBS	NM_000071.3	aggagaagtgtcctggatgc	taggttgtctgctccgtctg
CSE	NM_001902.6	gcatttcaaaaacggaatgg	ctcatgctgtggatgagagg
HO-1	NM_002133.3	tccgatgggtccttacactc	taaggaagcagcaagaga
MPST	NM_021126.8	acatcaaggagaacctggaatc	gatgtggccaggttcaatg
Nrf-2	NM_006164.5	gcaacaggacattgagcaag	tggacttggaaccatggtagt
RPLP13	NM_012423.4	caagcggatgaacaccaac	tgtggggcagcatacctc
YWHAZ	NM_003406.3	gatccccaatgcttcacaag	tgcttgttgtgactgatcgac

**Table 2:** Socio-demographic and clinical characteristics of serum donors. Columns in the middle show means of values ± SEM or percentage of population per each socio-demographic or clinical variable. Column on the right indicates the p-value obtained in the statistical test for a comparison of values for each variable between non DB-OA (n = 34) and DB-OA (n = 21) patients (*p* ≤ 0.05 shown in bold font, analysed by the Mann–Whitney test). * History of hypertension/dyslipidaemia (altered blood cholesterol and/or triglyceride levels).

	Non DB-OA Patients	DB-OA Patients	Statistical Differences
Sex (% women)	91.18	85.71	0.5422
Age (years)	64.82 ± 6.97	65.95 ± 8.88	0.9525
BMI (kg/m^2^)	28.21 ± 4.70	29.06 ± 4.03	0.4306
Obesity (%)	29.41	33.33	0.7702
Glucose levels (mg/dL)	85.96 ± 8.90	130.5 ± 32.80	**<0.0001**
Hyperglycaemia (glucose ≥110 mg/dL) (%)	0	82.35	-
Hypertension * (%)	41.18	61.90	0.1415
Body fat (%)	41.08 ± 9.05	42.66 ± 9.95	0.3451
Total cholesterol (mg/dL)	200.40 ± 29.65	188.30 ± 41.30	0.3349
Hypercholesterolemia (cholesterol ≥220 mg/dL) (%)	25.93	29.41	0.8148
Triglycerides (mg/dL)	101.90 ± 33.41	132.90 ± 61.17	0.0764
Dyslipidaemia * (%)	52.94	80.95	**0.0386**

**Table 3:** Socio-demographic and clinical characteristics of cartilage donors. Columns in the middle show means of values ± SEM or percentage of population per each socio-demographic or clinical variable. Column on the right indicates p-value obtained in the statistical test for a comparison of values for each variable between non DB-OA (n = 14) and DB-OA patients (n = 10) (*p* ≤ 0.05 shown in bold, analysed by the Mann–Whitney test). * History of hypertension/dyslipidaemia (altered blood cholesterol and/or triglyceride levels).

	non DB-OA Patients	DB-OA Patients	Statistical Differences
Sex (% women)	35.71	30.00	0.7815
Age (years)	71.36 ± 12.00	79.6 ± 9.73	0.1274
BMI (kg/m^2^)	30.17 ± 6.24	30.24 ± 5.99	0.9860
Obesity (%)	44.44	60.00	0.6110
Glucose levels (mg/mL)	98.79 ± 18.71	183.9 ± 80.88	**0.0009**
Hyperglycaemia (glucose ≥110 mg/dL) (%)	21.43	80.00	**0.003**
Hypertension * (%)	50.00	80.00	0.1466
Total Cholesterol (mg/dL)	150.20 ± 58.17	128.10 ± 31.67	0.2884
Hypercholesterolemia (cholesterol ≥220 mg/dL) (%)	14.29	0.00	-
Triglycerides (mg/dL)	155.70 ± 86.12	131.40 ± 37.67	0.4135
Hypertriglyceridemia (triglycerides ≥200 mg/dL) (%)	28.57	0.00	-
Dyslipidaemia * (%)	64.29	80.00	0.4259

**Table 4:** Treatments received by donors of serum and cartilage samples. Second and third columns from the left show the percentage of the study population having each treatment. Second column on the right indicates p-value obtained in the statistical test for the comparison of percentage of individuals having each treatment between non DB-OA and DB-OA patients. First column on the right shows p-value obtained in the statistical test for a comparison of the means of serum H_2_S or CBS/CSE expression between the treated and not treated patients. *p* ≤ 0.05 shown in bold, analysed by the Mann–Whitney test.

**Treatment for Serum Donors**	**Non DB-OA Patients** **n = 34**	**DB-OA Patients** **n = 21**	**Comparison OA-DB vs. OA-Non DB Patients**	**Comparison Treated vs. Non Treated (Serum H_2_S Levels)**
Hypertension	36.84%	65.22%	**0.0336**	0.4614
Hypercholesterolemia	44.74%	73.91%	**0.0281**	0.5516
Glucocorticoids	7.89%	4.35%	0.6027	-
Hypothyroidism	5.26%	8.70%	0.6148	-
Osteoporosis	21.05%	26.06%	0.6605	-
Anti-aggregation/anti-coagulants	7.89%	17.39%	0.2691	-
Methotrexate	18.42%	8.70%	0.3090	-
Anti-inflammatory	15.80%	21.74%	0.2844	-
**Treatment for Cartilage Donors**	**Non DB-OA Patients** **n = 14**	**DB-OA Patients** **n = 10**	**Comparison OA-DB vs. OA-Non DB Patients**	**Comparison Treated vs. Non Treated (CBS/CSE)**
Hypertension	43.75%	54.55%	0.6081	-
Hypercholesterolemia	56.25%	81.82%	0.1840	-
Glucocorticoids	6.25%	0.00%	-	-
Osteoporosis	6.25%	0.00%	-	-
Anti-aggregation/anti-coagulants	37.50%	90.91%	**0.0071**	0.3502/0.1195
Gout	18.75%	0.00%	-	-
Methotrexate	6.25%	0.00%	-	-

## Data Availability

The data used to support the findings of this study are contained within the article. Raw data are available from the corresponding author upon request.
